# Local complement activation is associated with primary graft dysfunction after lung transplantation

**DOI:** 10.1172/jci.insight.138358

**Published:** 2020-09-03

**Authors:** Hrishikesh S. Kulkarni, Kristy Ramphal, Lina Ma, Melanie Brown, Michelle Oyster, Kaitlyn N. Speckhart, Tsuyoshi Takahashi, Derek E. Byers, Mary K. Porteous, Laurel Kalman, Ramsey R. Hachem, Melanie Rushefski, Ja’Nia McPhatter, Marlene Cano, Daniel Kreisel, Masina Scavuzzo, Brigitte Mittler, Edward Cantu, Katrine Pilely, Peter Garred, Jason D. Christie, John P. Atkinson, Andrew E. Gelman, Joshua M. Diamond

**Affiliations:** 1 Department of Medicine, Washington University School of Medicine, St. Louis, Missouri, USA.; 2 Department of Medicine, Perlman School of Medicine at the University of Pennsylvania, Philadelphia, Pennsylvania, USA.; 3 Department of Surgery, Washington University School of Medicine, St. Louis, Missouri, USA.; 4 Barnes-Jewish Hospital, St. Louis, Missouri, USA.; 5 Department of Surgery, Perlman School of Medicine at the University of Pennsylvania, Philadelphia, Pennsylvania, USA.; 6 Laboratory of Molecular Medicine, Department of Clinical Immunology, Section 7631, Rigshospitalet and Faculty of Health and Medical Sciences, University of Copenhagen, Denmark.; 7 Department of Pathology and Immunology, Washington University School of Medicine, St. Louis, Missouri, USA.

**Keywords:** Pulmonology, Complement, Organ transplantation

## Abstract

**BACKGROUND:**

The complement system plays a key role in host defense but is activated by ischemia/reperfusion injury (IRI). Primary graft dysfunction (PGD) is a form of acute lung injury occurring predominantly due to IRI, which worsens survival after lung transplantation (LTx). Local complement activation is associated with acute lung injury, but whether it is more reflective of allograft injury compared with systemic activation remains unclear. We proposed that local complement activation would help identify those who develop PGD after LTx. We also aimed to identify which complement activation pathways are associated with PGD.

**METHODS:**

We performed a multicenter cohort study at the University of Pennsylvania and Washington University School of Medicine. Bronchoalveolar lavage (BAL) and plasma specimens were obtained from recipients within 24 hours after LTx. PGD was scored based on the consensus definition. Complement activation products and components of each arm of the complement cascade were measured using ELISA.

**RESULTS:**

In both cohorts, sC4d and sC5b-9 levels were increased in BAL of subjects with PGD compared with those without PGD. Subjects with PGD also had higher C1q, C2, C4, and C4b, compared with subjects without PGD, suggesting classical and lectin pathway involvement. Ba levels were higher in subjects with PGD, suggesting alternative pathway activation. Among lectin pathway–specific components, MBL and FCN-3 had a moderate-to-strong correlation with the terminal complement complex in the BAL but not in the plasma.

**CONCLUSION:**

Complement activation fragments are detected in the BAL within 24 hours after LTx. Components of all 3 pathways are locally increased in subjects with PGD. Our findings create a precedent for investigating complement-targeted therapeutics to mitigate PGD.

**FUNDING:**

This research was supported by the NIH, American Lung Association, Children’s Discovery Institute, Robert Wood Johnson Foundation, Cystic Fibrosis Foundation, Barnes-Jewish Hospital Foundation, Danish Heart Foundation, Danish Research Foundation of Independent Research, Svend Andersen Research Foundation, and Novo Nordisk Research Foundation.

## Abstract 2

Substantial differences between local and systemic complement activation in lung transplant recipients who develop primary graft dysfunction are identified in two independent cohorts.

## Graphical abstract

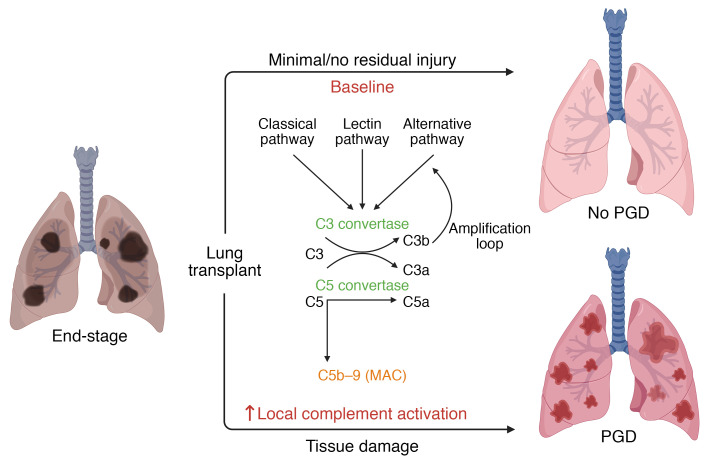

## Introduction

Primary graft dysfunction (PGD), a form of acute lung injury after lung transplantation (LTx) (1, 2), occurs in 25%–30% of patients after lung transplant. This complication leads to an increased duration of mechanical ventilation and hospitalization and an increased risk of bronchiolitis obliterans syndrome (3, 4). Although the mechanisms driving the development of PGD are not completely understood, ischemia/reperfusion injury (IRI) has been identified as the predominant inciting etiology (5, 6).

IRI initiates PGD by engaging pathways associated with inflammation and innate immune activation (6–9). We previously demonstrated that elevated plasma levels of the innate immune protein long pentraxin-3 (PTX3), produced by macrophages, dendritic cells, and endothelial cells, were associated with PGD in patients with idiopathic pulmonary fibrosis (10). Furthermore, we identified an association between PTX3 single nucleotide polymorphisms and PGD after LTx (11). PTX3 release leads to activation of the complement cascade, which drives an amplification loop of innate immunity that exacerbates inflammation (12, 13). However, whether this activation occurs within the lung or peripherally is currently unknown.

The complement cascade can be activated via 3 distinct pathways: classical, lectin, and alternative (14). The classical pathway is engaged by antigen-antibody complexes as well as by short (i.e., CRP) and long pentraxins (i.e., PTX3). Lectin pathway activation is mediated by surface recognition of carbohydrates on pathogens and injured tissues, such as lectins, ficolins, and collectins (13). Alternative pathway activation constitutively occurs at low levels and is amplified when C3 and factor B engage in a feedback loop to deposit C3b on a target (14). Activation of the complement system amplifies inflammation by generating anaphylatoxins (i.e., C3a, C5a), which increase blood vessel permeability and mediate leukocyte chemotaxis (14). Additionally, complement activation results in formation of membrane attack complexes (C5b-9) on endothelial cells, which have been implicated in acute lung injury (15, 16) as well as end-organ damage in different preclinical thoracic organ transplant models (17). We previously demonstrated that an increase in plasma C5a between 6 and 24 hours after transplantation was associated with severe PGD and an increased risk of death (18). C3d and C4d staining were also observed within 3 months after transplantation in lung allografts of subjects who developed severe PGD (19). Moreover, in a randomized, double-blinded, multicenter trial, prereperfusion administration of TP10 (a drug that reduced complement activation by inactivating the C3 and C5 convertases) decreased the duration of mechanical ventilation after LTx compared with placebo (20). Thus, complement activation appears to play an important role in PGD; however, if markers of complement activation differ in those who develop PGD versus those who do not, which pathways are engaged, and, importantly, whether this activation is systemic or primarily local, remain unanswered.

Here we sought to (a) assess whether markers of complement activation are elevated in the lung and blood compartments early after LTx in subjects with PGD, (b) identify which pathways for complement activation are engaged in these subjects, and (c) determine the relationship between complement activation in the bronchoalveolar lavage (BAL) and that in the systemic circulation.

## Results

### Demographic characteristics of the cohort.

**Figure 1:**
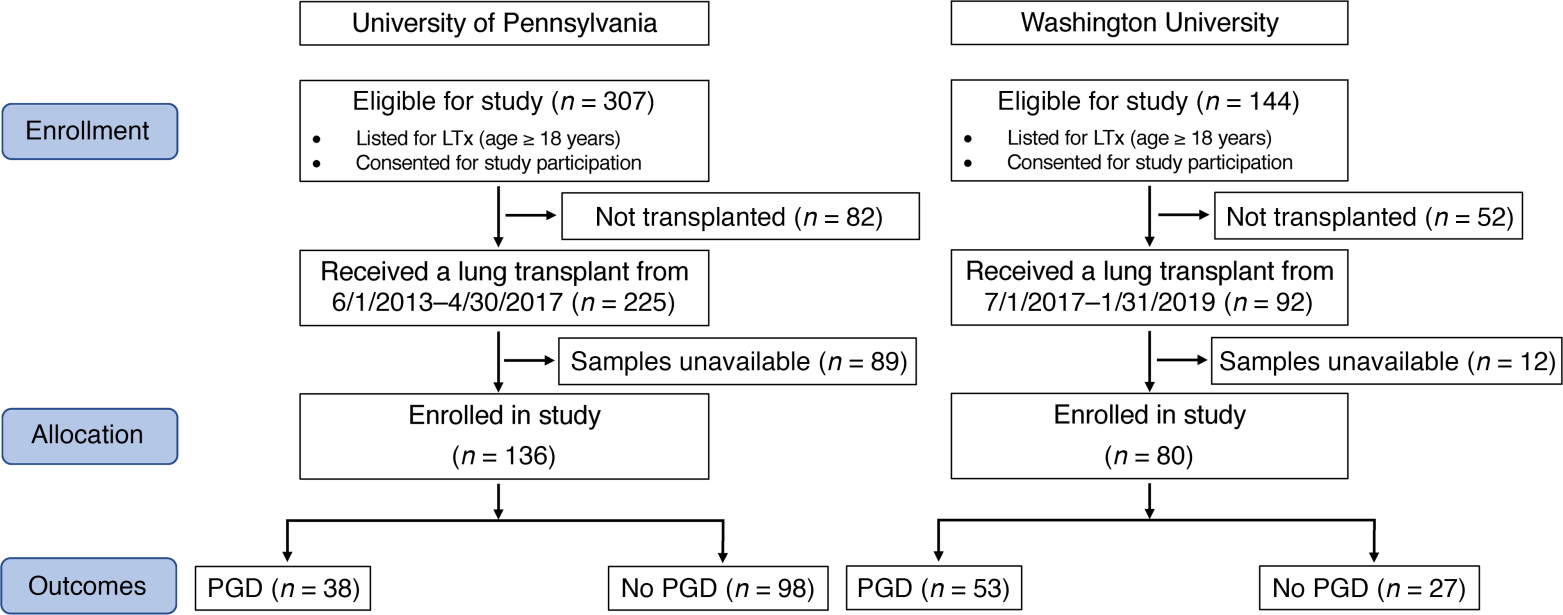
CONSORT flow diagram. CONSORT flow diagram for subjects enrolled in the study at the University of Pennsylvania and Washington University School of Medicine. LTx, lung transplantation; PGD, primary graft dysfunction.

In the Penn cohort (*n* = 136, Table 1 and Figure 1), the incidence of grade 3 PGD (Supplemental Tables 1 and 2; supplemental material available online with this article; https://doi.org/10.1172/jci.insight.138358DS1) between 48 and 72 hours was 27.9%, compared with that in the WUSM cohort (*n* = 80, Table 1 and Figure 1), in which it was only 6.3%. As a result, we reported results in those who developed any grade of PGD in the WUSM cohort (*n* = 53, 66.3%) and did subgroup analyses in (a) those who developed any grade of PGD at or after 24 hours (*n* = 44, 55%) and (b) those who developed grade 2 or 3 PGD at any time point (*n* = 29, 36.3%). The distribution of certain key factors, including intraoperative extracorporeal life support (ECLS), were more similar across centers when using these distinct PGD definitions to compare the 2 cohorts (as shown in Table 1).

### Complement activation is associated with increased PGD severity within the first 24 hours of LTx.

**Figure 2:**
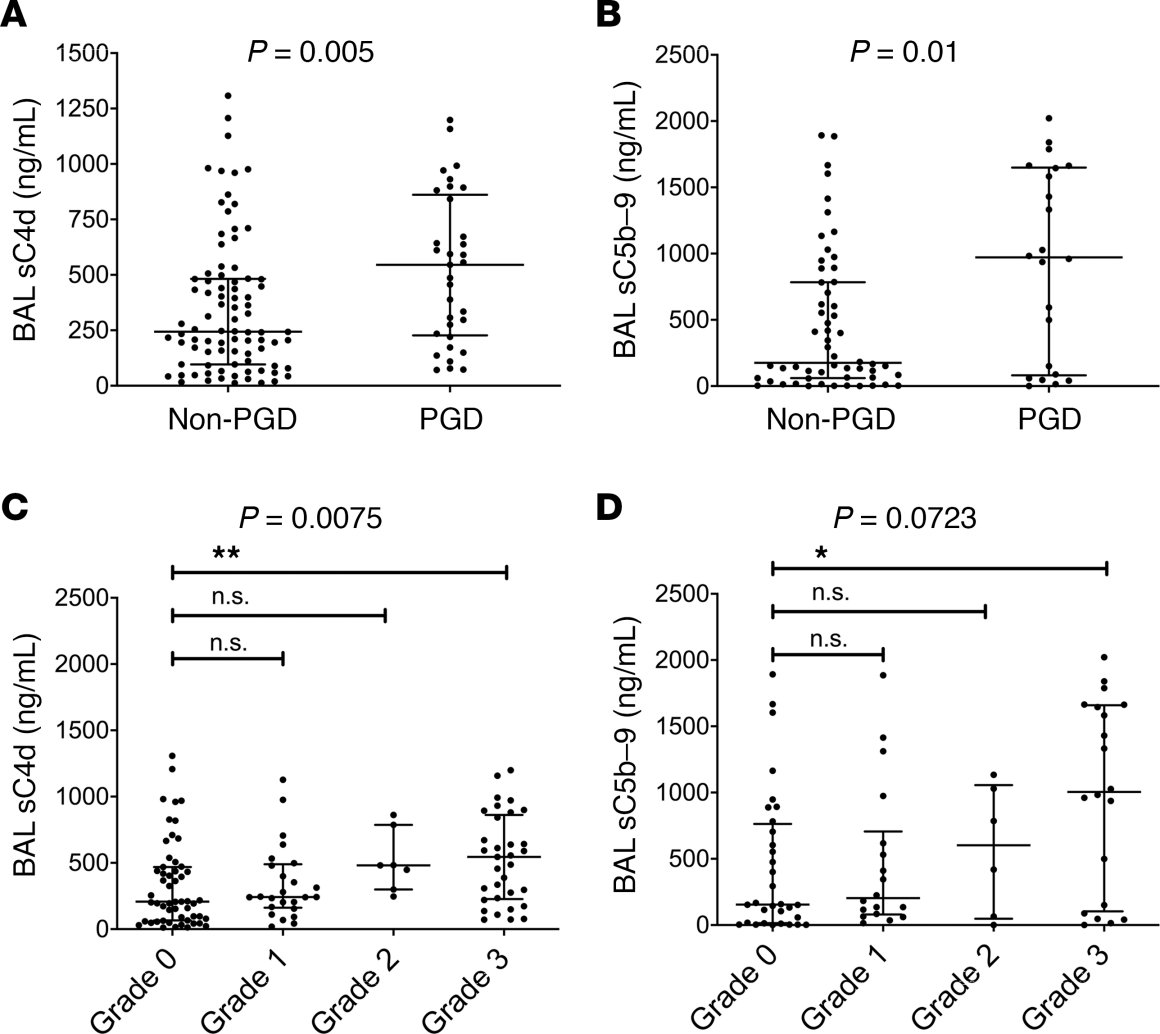
Complement activation is associated with increased PGD severity. Levels of sC4d (**A**) and sC5b-9 (**B**) in subjects who developed PGD compared with those who did not. Graphs in **C** and **D** demonstrate that the values of both sC4d and sC5b-9 increased as the PGD severity worsened. **P < 0.01 for grade 3 PGD vs. grade 0 PGD for sC4d, *P < 0.05 for sC5b-9; Mann-Whitney *U* test for **A** and **B** and Kruskal-Wallis test after adjusting for multiple comparisons (Dunn’s multiple comparisons test) for **C** and **D**. Penn cohort, *n* = 136.

The accumulation of C4d fragments, on tissue or in the fluid phase (as soluble C4d [sC4d]), or both, is a general marker of complement activation (21). In the Penn cohort, BAL concentrations of sC4d were higher in subjects with PGD (545.01 ng/mL) compared with subjects without PGD (243.97 ng/mL) (*P* = 0.005; Figure 2A and Table 2). sC4d levels remained higher in those with grade 2 or 3 PGD versus those with grade 0 or 1 PGD at any time point (*P* = 0.0006; Supplemental Figure 1A and Table 2). Thus, we identified differences in the accumulation of C4d fragments in the BAL of subjects with PGD in the Penn cohort, using 2 distinct definitions of PGD: (a) as normally reported in prior publications (1–4) (grade 3 in the first 48–72 hours) and (b) similar to the outcome definition as the WUSM cohort — grade 2 or 3 PGD at any time point. Additionally, the generation of the terminal complement complex (TCC), measured using BAL sC5b-9 levels, was higher in subjects with PGD (971.5 ng/mL) compared with subjects without PGD (175 ng/mL) (*P* = 0.01; Figure 2B, Supplemental Figure 1B, and Table 2) and highly correlated with concurrently measured BAL C4d levels (Spearman’s rho = 0.79, *n* = 77, *P* < 0.001). Both BAL sC4d and sC5b-9 levels progressively increased with the grade of PGD severity (*P* = 0.0075 for sC4d, *P* = 0.0723 for sC5b-9; Figure 2, C and D).

**Figure 3:**
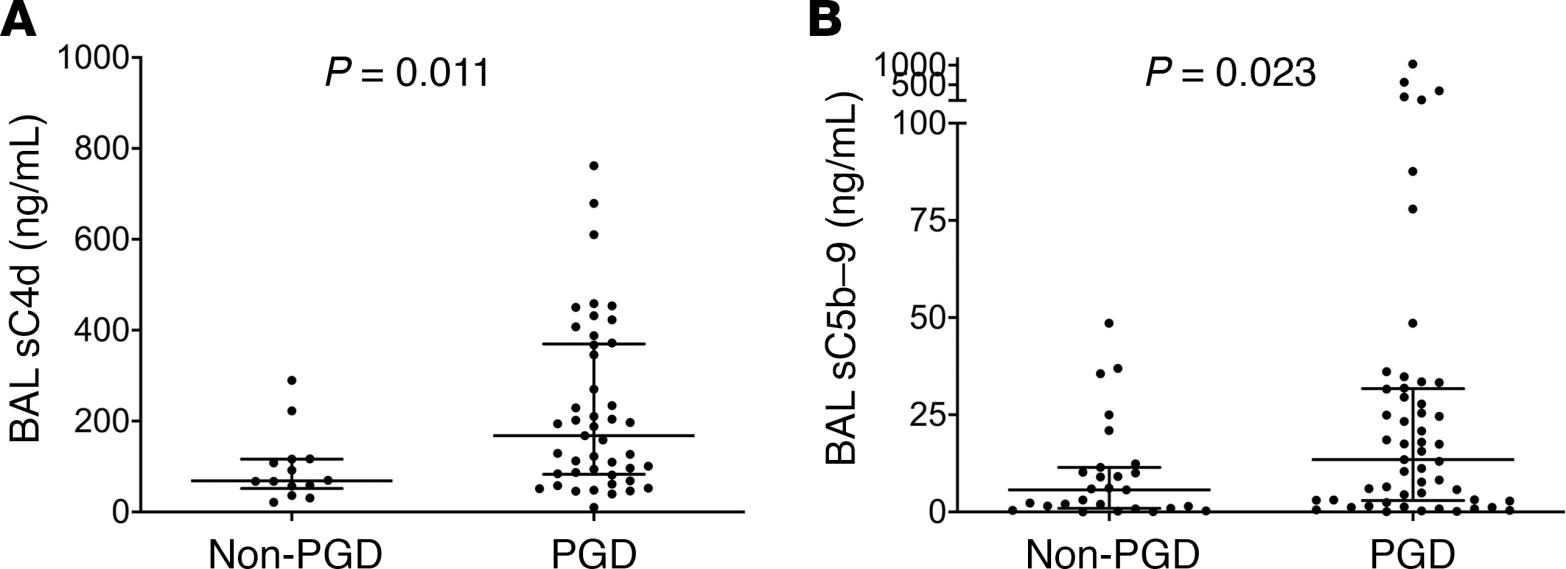
Complement activation is associated with increased PGD severity within the first 24 hours of lung transplantation. Levels of sC4d (**A**) and sC5b-9 (**B**) were elevated in subjects who developed PGD compared with those who did not. Note that there were inherent differences in clinical practices at the 2 centers, including the variability in the volume of fluid instilled into the lung, as well as the return, which partially explain the differences in the levels of sC4d and sC5b-9 when compared with Figure 2. Rank sum tests of comparison (Mann-Whitney *U* test). WUSM cohort, *n* = 80.

We then sought to test the results in a second independent cohort with inherent differences in PGD grades, lesser ECLS use, and later sampling points. Even in the WUSM cohort, median BAL concentrations of sC4d were higher in subjects with PGD (167.9 ng/mL) compared with subjects without PGD (68.82 ng/mL) (*P* = 0.011, Figure 3A and Table 2). Similar to the Penn cohort results, median sC5b-9 levels were higher in subjects with PGD (13.48 ng/mL) compared with subjects without PGD (5.69 ng/mL) (*P* = 0.023, Figure 3B and Table 2). This relationship held true when PGD was restricted to that occurring at or after 24 hours (*P* = 0.054, Supplemental Figure 2A). There was a difference between subjects with grade 2 or 3 PGD versus those with grade 0 or 1 PGD, but this did not reach statistical significance (*P* = 0.073 for sC5b-9, Supplemental Figure 2B and P** = 0.052 for sC4d, Supplemental Figure 2C). Levels of the central components of the complement cascade, such as C5 and C9, were elevated in the BAL of subjects with PGD, compared with that of subjects without PGD (Supplemental Figure 3, A and B, and Table 3). Additionally, soluble iC3b, which is generated upon complement cascade activation from C3, was elevated in the BAL of subjects with PGD compared with that from subjects without PGD. In particular, iC3b levels were higher in those subjects developing PGD (4822.53 ng/mL) compared with subjects without PGD (1359.67 ng/mL) (*P* = 0.006, Supplemental Figure 3C and Table 3). We also evaluated the iC3b/C3 ratio as a relative measure of complement activation; it was elevated in those subjects developing PGD (125.64 vs. 33.58, *P* = 0.009, Supplemental Figure 3D and Table 3). Finally, levels of BAL sC5b-9 highly correlated with concurrently measured BAL sC4d levels (Spearman’s rho = 0.694, *n* = 59, *P* < 0.001) and BAL iC3b levels (Spearman’s rho = 0.523, *n* = 36, *P* = 0.001).

Given that ECLS in itself is known to increase complement activation products (22, 23), we analyzed their levels in subjects (from both cohorts) who received ECLS compared with those who did not. In the Penn cohort, the median sC4d levels were comparable (median, interquartile range [IQR, ng/mL]: 365, 201.2–624.8 in those who received ECLS, compared with 241.2: 111–590.1 in those who did not, *P* = 0.3) as were the median sC5b-9 levels (median, IQR, ng/mL: 499, 114–1027 in those who received ECLS, compared with 294, 60–960 in those who did not, *P* = 0.5). These findings were replicated in the WUSM cohort, in which the median C4d levels were comparable (median, IQR, ng/mL: 112.64, 61.32–345.83, in those who received ECLS, compared with 119.73, 67.46–261.39, in those who did not, *P* = 0.78), as were the median sC5b-9 levels (median, IQR, ng/mL: 6.47, 1.19–25.53, in those who received ECLS, compared with 10.05, 1.99–25.44, in those who did not, *P* = 0.58).

### PGD severity is associated with the classical/lectin pathways of the complement cascade and activation of the alternative pathway.

We then sought to identify which pathways of complement activation were associated with PGD in the WUSM cohort. To this end, we measured C1q (a component of the classical pathway), mannose-binding lectin (MBL, a component of the lectin pathway), and C2 and C4 (components common to both the classical and lectin pathways), as well as factor B and factor D (components of the alternative pathway). We also measured C4b, which is cleaved from C4, pointing to classical and/or lectin pathway activation, and Ba, which is cleaved from factor B, indicating alternative pathway activation.

**Figure 4:**
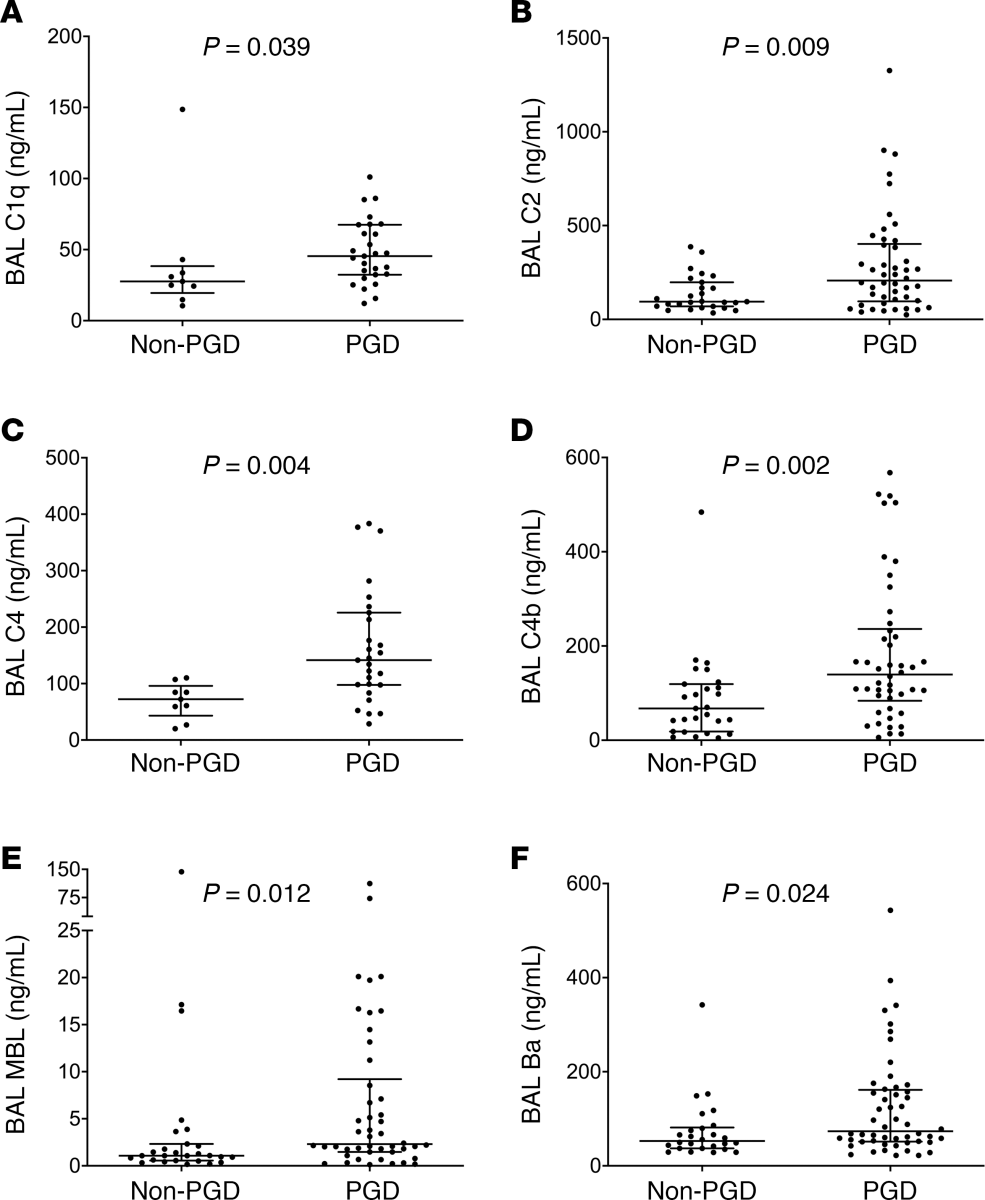
PGD severity is associated with multiple pathways of complement activation. Multiplex assays done in the WUSM cohort (*n* = 73, Table 3 and Supplemental Table 3) were used to compare bronchoalveolar lavage (BAL) fluid levels of C1q, (**A**), C2 (**B**), and C4 (**C**) in subjects who developed PGD compared with those who did not. The presence of C4b, suggestive of activation of both classical and lectin pathways, was compared in subjects with PGD and those without it (**D**). C2 and C4 are involved in both the classical and lectin pathways of complement activation, while C1q is specific to the classical pathway, and MBL (**E**) is specific to the lectin pathway. Additionally, Ba, which is generated from factor B and represents activation of the alternative pathway, was measured (**F**). Rank sum tests of comparison (Mann-Whitney *U* test).

We found that the median levels of C1q, C2, and C4 in the BAL were all elevated in subjects with PGD as compared with those without PGD (Figure 4, A–C, and Table 3). Additionally, median C4b levels were also elevated in the BAL of those subjects with PGD (139.59 ng/mL) compared with those without PGD (67.49 ng/mL), suggesting activation of both the classical and lectin pathways, not just an increase in their individual components (*P* = 0.002, Figure 4D and Table 3). Median MBL levels were also elevated in subjects with PGD (2.3 ng/mL) compared with subjects without PGD (1.08 ng/mL, *P* = 0.012 for PGD vs. subjects without PGD, Figure 4E and Table 3). Although we noted no differences in the absolute levels of factor B among those subjects who developed PGD as compared with those who did not develop PGD (Supplemental Figure 4A), the median levels of Ba were significantly elevated in these subjects (Figure 4F and Table 3), and the levels of factor D demonstrated a trend toward significance (Supplemental Figure 4B and Table 3). Taken collectively, our findings suggest that not only are individual analytes belonging to the classical and lectin pathways of the complement cascade higher among subjects with PGD, but analytes indicating alternative pathway activation are also elevated in these subjects.

### Local markers of lectin pathway engagement distinguish subjects with PGD.

**Figure 5:**
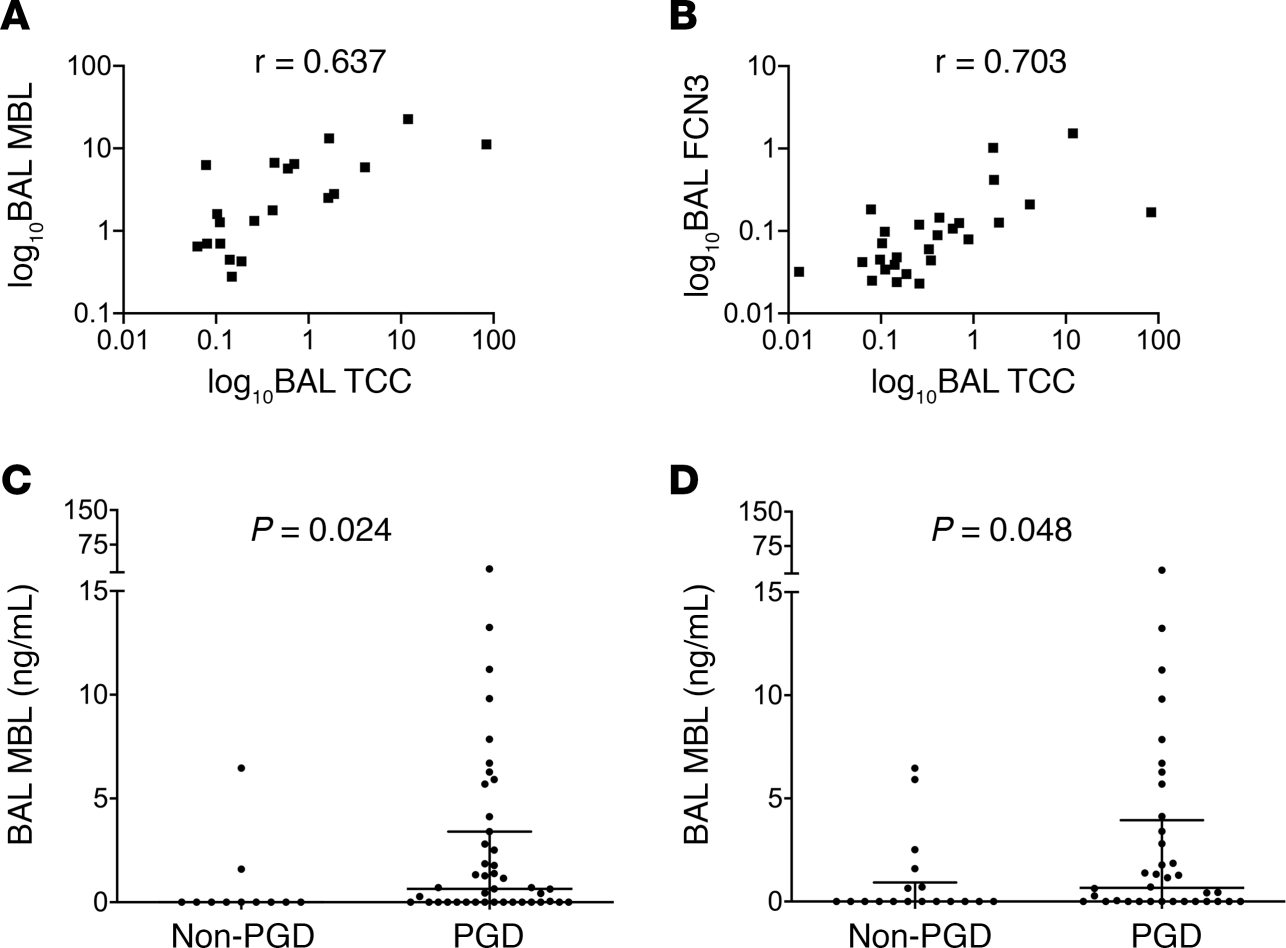
Local markers of lectin pathway activation distinguish subjects with PGD. Levels of mannose-binding lectin (MBL) in the bronchoalveolar lavage (BAL) highly correlated with markers of complement activation in the BAL (soluble terminal complement complex [TCC]) in the WUSM cohort (**A**, *n* = 40). Using a different assay than in Figure 4 (*n* = 73), BAL MBL levels were higher in subjects who developed PGD compared with the levels in subjects without PGD (**C**), and this held true in those who developed PGD at or after 24 hours (**D**). The levels of ficolin-3 (FCN3; **B**) also highly correlated with BAL TCC (*n* = 40). r represents Spearman’s rho coefficient. The axes were expressed in a logarithmic scale for purposes of graphical representation. Rank sum tests of comparison (Mann-Whitney U test).

Given the considerable variability in MBL levels in humans (24), we first quantified MBL in the BAL using a different assay and, second, did a comprehensive assessment of lectin pathway analytes in both the BAL and plasma levels in the WUSM cohort. We evaluated the association of these analytes with an independent marker of complement activation in both BAL and plasma, C4c, and validated it using a separate assay for sC5b-9 distinct from what we had used previously (termed TCC to semantically distinguish it from the prior assay). We found that BAL MBL levels highly correlated with markers of complement activation in the BAL (Spearman’s rho = 0.637 with TCC, Table 4 and Figure 5A, Spearman’s rho = 0.586 with C4c, Table 4 and Supplemental Figure 5A, both *P* < 0.001). Subjects with PGD had higher BAL MBL levels compared with those who did not develop PGD (Figure 5C and Supplemental Table 4). Additionally, in a subgroup analysis of those subjects who developed PGD at or after 24 hours, BAL MBL levels remained elevated in subjects who developed PGD compared with those who did not develop PGD (0.667 vs. 0.00, *P* = 0.048, Figure 5D).

Next, we probed for different proteins that engage with the lectin pathway. Among the ficolins, we found that BAL ficolin-3 (FCN-3) levels at 24 hours strongly correlated with markers of complement activation in the BAL (Spearman’s rho = 0.703 with TCC, Table 4 and Figure 5B, Spearman’s rho = 0.679 with C4c, Table 4 and Supplemental Figure 5B, both *P* < 0.001). Despite these differences in local complement activation products in LTx, there was minimal-to-no correlation between BAL TCC levels and plasma levels of TCC, C4c, or the other markers of lectin pathway engagement, nor were there any differences in the absolute levels (Supplemental Tables 5 and 6). There was also no correlation between the BAL levels of TCC and FCN-1 or FCN-2 (Table 4). These observations suggest that specific markers of the lectin pathway in the BAL highly correlate with evidence of local complement activation.

**Figure 6:**
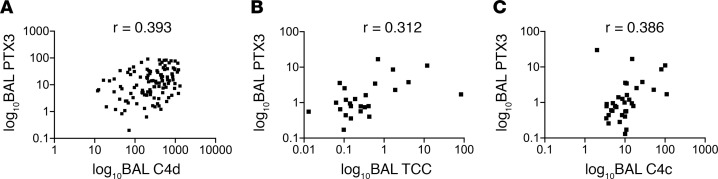
Local PTX3 moderately correlated with markers of complement activation. Levels of long pentraxin 3 (PTX3) in the bronchoalveolar lavage (BAL) only had a modest correlation with markers of complement activation in the BAL (C4d) in the Penn cohort (**A**, *n* = 113). This correlation held true in the second independent cohort (WUSM, *n* = 40) for the terminal complement complex (TCC; **B**) and was also true for C4c (**C**). r represents Spearman’s rho coefficient. The axes were expressed in a logarithmic scale for purposes of graphical representation.

Given that (a) there is a known association between plasma PTX3 levels and PGD in lung transplant recipients (10) and (b) PTX3 is associated with activation of both the classical/lectin pathways of the complement system (13), we further asked if PTX3 levels were associated with either circulating or local markers of complement activation in these subjects. In the Penn cohort, there was a modest positive correlation between BAL PTX3 levels with concurrently measured BAL sC4d levels (Spearman’s rho = 0.393, *P* < 0.001, Figure 6A). This relationship also held true in the WUSM cohort, in which we used independent assays. We found a modest correlation between BAL PTX3 levels and markers of complement activation (Spearman’s rho = 0.312 with TCC, Figure 6B, Spearman’s rho = 0.386 with C4c, Figure 6C, both *P* < 0.05) in the WUSM cohort, despite there being no correlation between plasma PTX3 levels and markers of complement activation (Spearman’s rho = –0.034) within 24 hours after LTx (Supplemental Table 5). Finally, there were also no differences in the median values of BAL PTX3 in subjects with PGD (15.18 ng/mL, *n* = 31) and subjects without PGD (12.99 ng/mL, *n* = 81) in the Penn cohort (*P* = 0.4, Supplemental Figure 6). These observations suggest there are components of the lectin pathway, other than PTX3, that better correlate with markers of complement activation and distinguish those subjects with PGD.

## Discussion

While the role of complement activation in acute lung injury has been evaluated in both experimental (16) and clinical settings (25, 26), its role in lung allograft injury is only beginning to be explored (27, 28). While targeting the complement cascade has been successful in certain preclinical models (29–31), follow-up studies have been met with tempered enthusiasm, despite moderate success in clinical trials (20, 32). This is likely due to a lack of data regarding (a) whether the complement cascade is actually being activated, as determined by formation of the membrane attack complex (vs. detecting anaphylatoxins generated by serine proteases i.e., elastase), (b) at what time point after LTx it is activated, and (c) if this activation occurs in the lungs. Additionally, we do not know which pathways to target, given that the complement cascade can be activated via multiple routes. For example, C1-esterase inhibitor — the therapeutic that has shown the most promise in human lung transplant recipients (32) — has multiple targets, including several that extend beyond inhibiting complement activation (33). Hence, given the increasing data on the role of complement activation in human LTx (18, 19), answering the above-mentioned questions is critical before developing targeted therapeutics for mitigating PGD.

Using 2 independent cohorts, we demonstrated that complement activation occurs primarily in the lungs (assessed using BAL fluid), as compared with that detected in the circulation of subjects with PGD within the first 24 hours after LTx. We demonstrated the specificity of this activation using soluble C5b-9 (sC5b-9), which is reflective of the membrane attack complex or TCC being formed due to complement cascade activation. We also identified complement activation using multiple independent markers (i.e.,iC3b, C4b, C4d). Using a panel of analytes to differentiate components of the classical, lectin, and alternative pathways, we showed that C4 (as well as C4b, indicating cleavage of C4), C1q, and MBL are higher in subjects with PGD compared with those without PGD, as are Ba levels, suggesting alternative pathway activation. We also detected higher levels of C2 in the BAL of subjects with PGD, and alveolar epithelial cells are a known source of C2 production (14). These findings show complement activation within grafts damaged by IRI is primarily occurring through classical and/or lectin pathway activation but likely amplified by the alternative pathway (34, 35). We also used 2 independent measures of MBL levels in the BAL of LTx recipients to further validate our findings.

These findings prompt the question of what could be activating the classical or lectin pathway in subjects with PGD. The classical pathway is primarily activated by immune complexes comprising antigens bound to specific antibodies that can fix C1q, resulting in formation of the C1 complex (36). Formation of this complex activates the serine protease C1s, which can then trigger a cascade involving the sequential cleavage of C4, followed by C2, then C3, and C5, eventually resulting in the formation of C5b-9 on the target surface. However, C1 also binds to C-reactive protein and PTX3, among other acute-phase reactants (37). Moreover, different lectins bind to the surface of carbohydrates to activate MBL/ficolin/collectin-associated serine proteases (MASPs), which are enzymes that can attach to substrates such as MBL and facilitate C2 and C4 cleavage (38). Additionally, FCN-3, which forms a substrate for binding to MASPs (39–41), was highly correlated with markers of complement activation in the WUSM cohort. FCN-3 is synthesized in the liver and bile ducts but also in the lungs (42). Additionally, tissue-specific recruitment of FCN-3 to the lungs has been reported in the context of LPS-mediated acute lung injury in humans (43). These observations led us to consider the possibility that increased airway accumulation of these proteins after IRI may be triggering local complement cascade activation in the lungs via the lectin pathway.

Although PTX3 has been associated with IRI in other model systems (44) and is a known activator of the complement cascade (12, 13), it has remained unclear if its accumulation correlates with markers of complement activation. Interestingly, the association of PTX3 with markers of complement activation in the BAL was modest and, unlike MBL, was not different in those subjects with and without PGD. Moreover, this correlation, being primarily observed in the BAL and not in the plasma in subjects with PGD, suggests that activated complement products are concentrated to sites of injury. Our data are consistent with our previous findings in patients with other forms of pulmonary inflammation in which the complement system is implicated, such as antibody-mediated rejection of the lung (28). Additionally, our observations create a precedent for evaluating how intragraft MBL activation in the setting of PGD could trigger chronic lung allograft dysfunction (CLAD). MBL localizes to the endothelium and basement membrane during cold ischemia and obliterative bronchiolitis (45). On the other hand, low MBL levels in the airway have also been associated with CLAD, presumably through decreased efferocytosis (46). Thus, testing if modulating the lectin pathway early after IRI reduces CLAD in experimental models of LTx may potentially lead to a more targeted therapeutic approach in reducing PGD.

Our study has several limitations. First, there was a considerable difference in the overall incidence of PGD between the 2 cohorts, likely driven by differences in the patient populations at the 2 centers. This has been previously reported in multicenter studies, wherein the range of severe PGD ranged from 2%–27% (47). However, the relationship between complement activation and PGD held true when restricting the analysis to PGD occurring after 24 hours and those having grade 2 or 3 PGD. Second, our sample size was small. A larger sample size of patients within our selected date range could not be gathered due to limits in the availability of concurrent plasma and BAL samples. Nevertheless, our sample size is consistent with other reported biomarker experiments in LTx (48–50). Third, we did not test for all the different MASPs, which would have provided additional specificity for the proteases that facilitate lectin pathway activation. Fourth, most studies using BAL bring into question whether the fluid is contaminated by blood/plasma, especially in the setting of an acute inflammatory process such as PGD. However, adjusting for permeability remains unreliable in human studies (51, 52). Additionally, the fact that there was no difference in these markers of activation in the plasma of subjects with and without PGD would suggest that these differences are specific to the BAL, not simply a measure of increased permeability. Fifth, there were inherent differences in clinical practices at the 2 centers, including the variability in the volume of fluid instilled into the lung as well as the return. However, in spite of these differences, the relationship between local complement activation and PGD was still evident in both cohorts, providing further support for our overall findings. Finally, in accordance with other studies, we cannot state a definitive relationship between higher BAL markers of complement activation and the increased PGD risk; rather, we can only describe the association between the 2 factors.

We conclude that there are marked differences between local and systemic complement activation in subjects who develop PGD. This difference was most evident in the local accumulation of not only the classical and lectin pathway components, but also activation fragments of the alternative pathway. MBL appears to be the most prominent marker of lectin pathway activation in subjects with PGD. Additionally, elevated levels of FCN-3 in the BAL highly correlate with markers of complement activation. Future studies will involve exploring the relationship between lung-specific and systemic innate immune activation and local targeting of the lectin pathway in experimental lung transplant models as a potential therapeutic avenue for reducing PGD incidence in patients with LTx.

## Methods

### Study design, settings, and participants

This retrospective cohort study used BAL samples that had been independently collected at 2 centers, the University of Pennsylvania and Washington University School of Medicine (Figure 1).

Participants were enrolled at the University of Pennsylvania as part of the Prospective Registry of Outcomes in Patients Electing Lung Transplantation (PROPEL) (Penn cohort) (47). In this cohort, all BAL samples were collected from patients receiving a lung transplant between June 2013 and April 2017 at the Hospital of the University of Pennsylvania. At approximately 2 hours after allograft reperfusion, 20 mL PBS was instilled into the right middle lobe of the lung, and a return of 5–10 mL was obtained, which was centrifuged, separated into supernatant aliquots and cell pellet suspensions, and then stored at –80°C until experimentation. Clinical data were collected for all patients.

BAL samples collected at Washington University School of Medicine between July 2017 and January 2019 (WUSM cohort) were used as a second independent cohort and for subsequent analyses. In this cohort, at 18–24 hours after allograft reperfusion, 100 mL PBS was instilled into the right middle lobe of the lung, centrifuged, separated into aliquots, and stored. The processing of the samples in the laboratory was similar to that at the University of Pennsylvania. Blood samples were collected from the same recipients at the same time the BAL specimens were obtained. These were transported on ice, centrifuged to collect plasma, and stored at –80°C.

### Outcome definition

PGD is defined as a syndrome of acute lung injury occurring early after LTx, graded on the basis of pulmonary edema on chest radiographs and the degree of hypoxemia, and measured by the PaO_2_/FiO_2_ ratio. PaO2 is the partial pressure of arterial oxygen, and FiO2 is the fraction of inspired oxygen (Supplemental Table 1) (1). In the Penn cohort, the primary outcome considered was grade 3 PGD occurring within the first 48–72 hours after LTx, as defined by the International Society for Heart and Lung Transplant guidelines (2). Because of institutional differences in recipient characteristics between the Penn and WUSM cohorts, including the difference between the number of transplants done on ECLS, the number of grade 2 or 3 PGD cases was fewer in the WUSM cohort. Because there were fewer cases of grade 2 or 3 PGD in the WUSM cohort, an alternative definition of “any grade of PGD (any PGD)” was used for the analysis in the WUSM cohort. In this scenario, patients with any grade of PGD (grades 1–3) were considered as having PGD, and a sensitivity analysis was conducted after excluding those who developed PGD before 24 hours. Additionally, a subgroup analysis was done considering the smaller number of subjects in the WUSM cohort who had grade 2 or 3 PGD. PGD scoring was independently done in a blinded manner by members of the transplant team (47).

### Protein concentration measurement

Participants were screened for complement activation in the BAL using the sC5b-9 assay (BD OptEIA Human C5b-9 ELISA set) and the sC4d assay (Quidel MicroVue ELISA). Individual complement analytes were evaluated using a modified MILLIPLEX MAP Human Complement Panel 1 and 2 (MilliporeSigma). Lectin pathway analytes were measured in both plasma and BAL. All BAL or plasma aliquots that were used had been through no more than 1 freeze-thaw cycle. Laboratory personnel were blinded to PGD status.

### Measurements of different complement components

#### sC5b-9 assay.

Participants were screened for complement activation in the BAL using the sC5b-9 assay (BD OptEIA Human C5b-9 ELISA set) (53, 54). Per the manufacturer’s instructions, purified native human C3, C4, C5, C6, C7, C8, and C9 have been tested in the BD OptEIA assay at ≥5 mg/mL, and no cross-reactivity (value ≥470 pg/mL) was identified.

#### sC4d assay.

sC4d was detected in the BAL using a highly specific C4d assay (Quidel MicroVue ELISA), which has previously been used to detect C4d in BAL fluid. (55, 56)

#### Individual complement analytes using MILLIPLEX assay.

Individual complement analytes were evaluated using a modified MILLIPLEX MAP Human Complement Panel 1 and 2 (MilliporeSigma, Supplemental Table 3) based on the Luminex xMAP technology, a bead-based multiplex assay. Specifically, we used the MILLIPLEX MAP Human Complement Panel 1 (HCMP1MAG) to simultaneously quantify the following analytes in the BAL: complement C2, complement C4b, complement C5, complement C5a, complement C9, adipsin (complement factor D), and MBL. Of note, this assay is specific for C4b and does not cross-react with C4d (per communication with manufacturer). The C5 measurements are distinct from C5a, as the intact factors are designed such as they would not detect individual fragments based on their capture and detection antibodies.

Similarly, we used the MILLIPLEX MAP Human Complement Panel 2 (HCMP2MAG) to simultaneously quantify the following analytes in the BAL: complement C1q, complement C3, complement C3b/iC3b, complement C4, and complement factor B. Of note, the assay for C3b/iC3b detects both C3b and iC3b (per communication with manufacturer). The factor B assay does not detect either fragments Bb or Ba.

#### Lectin pathway analytes.

The following lectin pathway pattern recognition molecules were measured in both plasma and BAL in duplicate: MBL, PTX3, FCN-1, FCN-2, and FCN-3 were quantified in specific sandwich ELISAs with monoclonal antibodies for each marker developed in the Laboratory of Molecular Medicine, Rigshospitalet, according to previously described procedures (57–61). All assays were optimized for automated analysis in a 384-well format on the Biomek FX robotic system (Beckman Coulter) (62). Alternative assays for C4 activation, i.e., C4c and sC5b-9 (TCC) were measured in ELISAs based on monoclonal antibodies against neoepitopes on the C4c fragment and C9 not exposed on the native molecules as described previously (63, 64).

#### Alternative pathway analytes.

Ba was measured in duplicate in the WUSM cohort using the Microvue Complement Ba fragment EIA kit (A033, Quidel Inc.).

#### PTX3 assay.

BAL concentrations of PTX3 were measured in duplicate in the Penn cohort using commercially available sandwich ELISAs (PTX3, Quantikine ELISA) as described previously (10, 11).

### Statistics

PGD associations with BAL levels of complement proteins were evaluated using rank sum tests. Given the sample size, we were stricter in our hypothesis testing and used nonparametric tests for comparison (e.g., assuming the data did not follow a normal distribution). Specifically, 2 independent groups were compared using the Mann-Whitney *U* test, and multiple group comparisons were done using nonparametric test for trends. For statistical analysis, the analysis corrected for multiple comparisons when comparing more than 2 groups. All statistical tests for comparison were 2 sided, and *P* < 0.05 was considered significant. In all figures, the error bars are defined such that data represent median with IQR. The correlation between complement activation proteins and individual analytes was assessed using Spearman’s correlation. Statistical analysis was performed using STATA 13.1 software (STATA Corp.); GraphPad Prism 8 was used for generating graphs.

### Study approval

The institutional review boards of University of Pennsylvania and Washington University School of Medicine independently approved this study, and written informed consent was obtained from all participants before inclusion in the study.

## Author contributions

HSK, JPA, JDC, AEG, and JMD provided study design. TT, BM, KNS, MB, MO, MR, and EC collected samples. LM, KR, HSK, MC, KP, PG, JM, and MC performed assays. HSK, LM, KR, LK, MKP, MC, MS, DEB, RRH, DK, JPA, JDC, AEG, JMD, KP, and PG provided data analysis and interpretation. HSK, AEG, and JMD drafted the manuscript. All authors provided critical revision and the final decision to submit.

## Supplementary Material

Supplemental data

ICMJE disclosure forms

## Figures and Tables

**Table 4:**
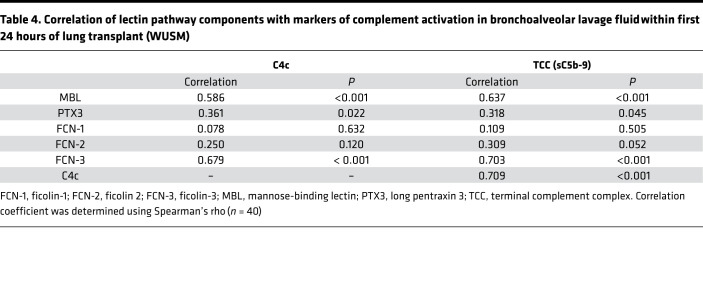
Correlation of lectin pathway components with markers of complement activation in bronchoalveolar lavage fluid within first 24 hours of lung transplant (WUSM)

**Table 1:**
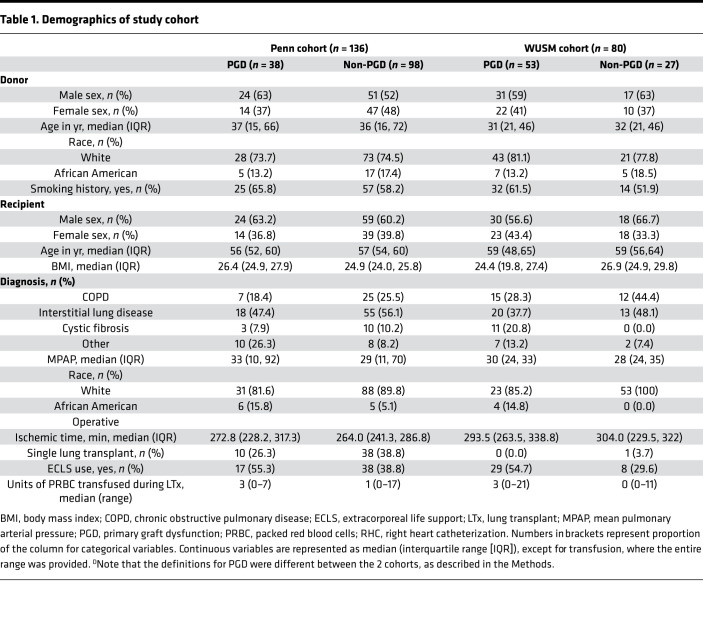
Demographics of study cohort

**Table 2:**
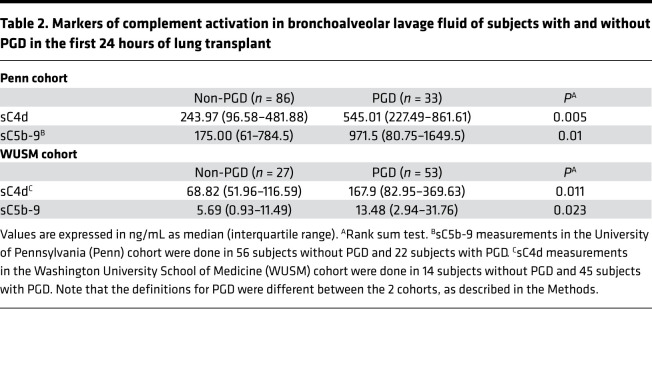
Markers of complement activation in bronchoalveolar lavage fluid of subjects with and without PGD in the first 24 hours of lung transplant

**Table 3:**
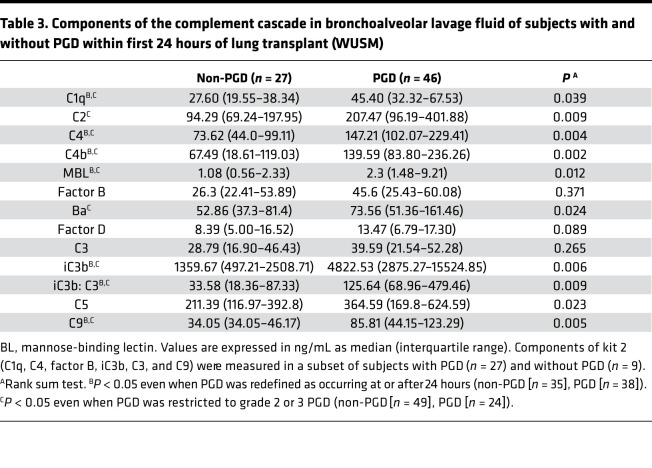
Components of the complement cascade in bronchoalveolar lavage fluid of subjects with and without PGD within first 24 hours of lung transplant (WUSM)

## Version 1 08/04/2020

In-Press Preview

## Version 2 09/03/2020

Electronic publication
