# Supplementary material for: Local complement activation is associated with primary graft dysfunction after lung transplantation
Source: JCI Insight. 2020 Sep 3;5(17):e138358. doi: 10.1172/jci.insight.138358 (PMC7526453; doi:10.1172/jci.insight.138358)
Supplement: Supplemental data [file jciinsight-5-138358-s138.pdf]

ONLINE SUPPLEMENT

Table S1. Grading of primary graft dysfunction (PGD)

Grade	Pulmonary edema on chest radiography	Degree of hypoxemia (PaO_2/FiO_2 ratio)
**PGD 0 (no PGD)**	**Absent**	**> 300**
**PGD 1 (mild)**	**Present**	**> 300**
**PGD 2 (moderate)**	**Present**	**200 – 300**
**PGD 3 (severe)**	**Present**	**< 200**

Adapted from Snell et al. J Heart Lung Transplant 2017 (1).
[a]If FiO_2 = 0.3 on nasal cannula or ventilator, if edema is present on x-ray, the subject is scored as having 'Grade 1', if edema is absent, it is 'Grade 0'.
[b]If subject is on extracorporeal lung support or mechanical ventilation with FiO_2 > 0.5 on nitric oxide at greater than 48 h after lung transplant, they are 'Grade 3'.
[c]PGD is graded at 4 time points, every 24 h, over the first 72 h after transplantation (6 h, 24 h, 48 h, 72 h).
[d]If multiple blood gas values are available, the worst PaO_2/FIO_2 ratio is used.
Abbreviations: FiO_2, fraction of inspired oxygen; PaO_2, partial pressure of arterial oxygen.

Table S2. Distribution of PGD scoring in the WUSM and Penn cohorts

	**WUSM cohort (n=80)**				**Penn cohort (n=136)**			
	**6 h**	**24 h**	**48 h**	**72 h**	**6 h**	**24 h**	**48 h**	**72 h**
**PGD 0**	39 (42)	42 (45)	46 (50)	54 (58)	**73 (54)**	**76 (56)**	**70 (51)**	**77 (57)**
**PGD 1**	17 (18)	26 (28)	24 (26)	20 (22)	**8 (6)**	**13 (10)**	**28 (21)**	**24 (18)**
**PGD 2**	8 (9)	7 (8)	8 (9)	2 (2)	**9 (7)**	**12 (9)**	**5 (4)**	**11 (8)**
**PGD 3**	16 (17)	5 (5)	2 (2)	4 (4)	**46 (34)**	**35 (26)**	**33 (24)**	**23 (17)**

[a]PGD is graded at 4 time points, every 24 h, over the first 72 h after transplantation (6 h, 24 h, 48 h, 72 h). 6 h: within 6 h of lung reperfusion. 24 h, 48 h and 72 h have time windows ± 6 h.
[b]Values in brackets represent the proportion of the total cohort (i.e. 80 in WUSM, 136 in Penn)

Table S3. Measurement of complement analytes reported in the study

Figure	Analyte	Kit
1	sC4d	Quidel® MicroVue C4d ELISA (A009)
	sC5b-9	BD OptEIA Human C5b-9 ELISA set (558315)
2	sC4d	Quidel® MicroVue C4d ELISA (A009)
	sC5b-9	BD OptEIA Human C5b-9 ELISA set (558315)
	iC3b	MILLIPLEX MAP Human Complement Panel 2 (HCMP2MAG-19K)
	C3	MILLIPLEX MAP Human Complement Panel 2 (HCMP2MAG-19K)
3	C1q	MILLIPLEX MAP Human Complement Panel 2 (HCMP2MAG-19K)
	C2	MILLIPLEX MAP Human Complement Panel 1 (HCMP1MAG-19K)
	C4	MILLIPLEX MAP Human Complement Panel 2 (HCMP2MAG-19K)
	C4b	MILLIPLEX MAP Human Complement Panel 1(HCMP1MAG-19K)
	MBL	MILLIPLEX MAP Human Complement Panel 1 (HCMP1MAG-19K)
	C5	MILLIPLEX MAP Human Complement Panel 1 (HCMP1MAG-19K)
	C9	MILLIPLEX MAP Human Complement Panel 1 (HCMP1MAG-19K)
	Factor B	MILLIPLEX MAP Human Complement Panel 2 (HCMP2MAG-19K)
	Factor D	MILLIPLEX MAP Human Complement Panel 1 (HCMP1MAG-19K)
	Ba	Quidel® MicroVue Ba Fragment EIA (A033)
4	MBL	Custom (Laboratory of Molecular Medicine, Rigshospitalet)
	FCN-1, FCN-2, FCN-3	Custom (Laboratory of Molecular Medicine, Rigshospitalet)
	C4c	Custom (Laboratory of Molecular Medicine, Rigshospitalet)
	sC5b-9/TCC	Custom (Laboratory of Molecular Medicine, Rigshospitalet)
5	PTX3 (Penn)	R&D Quantikine® ELISA (DPTX30)
	sC4d (Penn)	Quidel® MicroVue C4d ELISA (A009)

	PTX3 (WUSM)	Custom (Laboratory of Molecular Medicine, Rigshospitalet)
	C4c (WUSM)	Custom (Laboratory of Molecular Medicine, Rigshospitalet)
	TCC (WUSM)	Custom (Laboratory of Molecular Medicine, Rigshospitalet)

Abbreviations: FCN-1: ficolin-1; FCN-2: ficolin-2; FCN-3: ficolin-3; MBL: mannose-binding lectin; PTX3: long pentraxin-3; TCC: terminal complement complex.

Table S4. Components of the lectin pathway in bronchoalveolar lavage fluid of subjects with and without PGD within first 24 h of lung transplant.

	**Non-PGD (n=11)**	**PGD (n=43)**	**p**
**MBL***	**0.00 (0.00 – 0.00)**	**0.65 (0.00 – 3.41)**	**0.024**
PTX3	0.80 (0.27 – 1.57)	0.89 (0.44- 1.79)	0.725
FCN-1	6.84 (3.43 – 20.40)	7.50 (3.03 – 17.90)	0.991
FCN-2	0.00 (0.00 – 0.00)	0.00 (0.00 – 0.00)	0.166
FCN-3	0.044 (0.022 – 0.07)	0.06 (0.03 – 0.15)	0.201
C4c	7.13 (4.83 – 14.11)	10.10 (4.15 – 14.33)	0.813

Values are expressed in ng/ml as median (interquartile range). p reported on comparison using rank-sum test. *indicates that $p < 0.05$ even when PGD was redefined as occurring at or after 24 h [non-PGD (n=20), PGD (n=33)].

Abbreviations: FCN-1: ficolin-1; FCN-2: ficolin-2; FCN-3: ficolin-3; MBL: mannose-binding lectin; PTX3: long pentraxin-3

Table S5. Correlation of lectin pathway components in the plasma with markers of complement activation within first 24 h of lung transplant.

	Plasma C4c		Plasma TCC (sC5b-9)		BAL TCC (sC5b-9)	
	**Correlation**	**p**	**Correlation**	**p**	**Correlation**	**p**
MBL*	0.032	0.800	0.158	0.215	0.153	0.380
PTX3	0.062	0.627	0.201	0.115	0.223	0.198
FCN-1	0.142	0.266	0.108	0.402	-0.328	0.055
FCN-2	0.192	0.131	-0.19	0.353	-0.273	0.112
FCN-3	0.034	0.791	0.143	0.268	-0.087	0.620
C4c	-	-	0.266	0.035	-0.236	0.173

Correlation coefficient is determined using Spearman's rho for plasma (n=63) and for bronchoalveolar lavage (BAL, n=35). Plasma samples were obtained at the same time as the BAL specimens reported in this table and Table 4.

Abbreviations: FCN-1: ficolin-1; FCN-2: ficolin-2; FCN-3: ficolin3; MBL: mannose-binding lectin; PTX3: long pentraxin-3; TCC: terminal complement complex.

Table S6. Absolute levels of lectin pathway components in the plasma of subjects within first 24 h of lung transplant.

	Non-PGD (n=14)	PGD (n=39)	p
**MBL***	**411.88 (262.19 – 746.61)**	**751.1 (315.37 – 1478.71)**	**0.094**
PTX3	78.91 (38.71 – 94.14)	84.21 (54.61 – 145.06)	0.170
FCN-1	148.59 (111.81 – 204.60)	138.46 (98.18 – 191.95)	0.614
FCN-2	4.17 (3.25 – 6.93)	4.08 (3.30 – 5.49)	0.920
FCN-3	13.35 (10.63 – 16.08)	12.88 (10.50 - 15.58)	0.920
C4c	548.99 (352.05 – 676.40)	510.73 (410.95 – 663.58)	0.952

Supplemental Figure 1

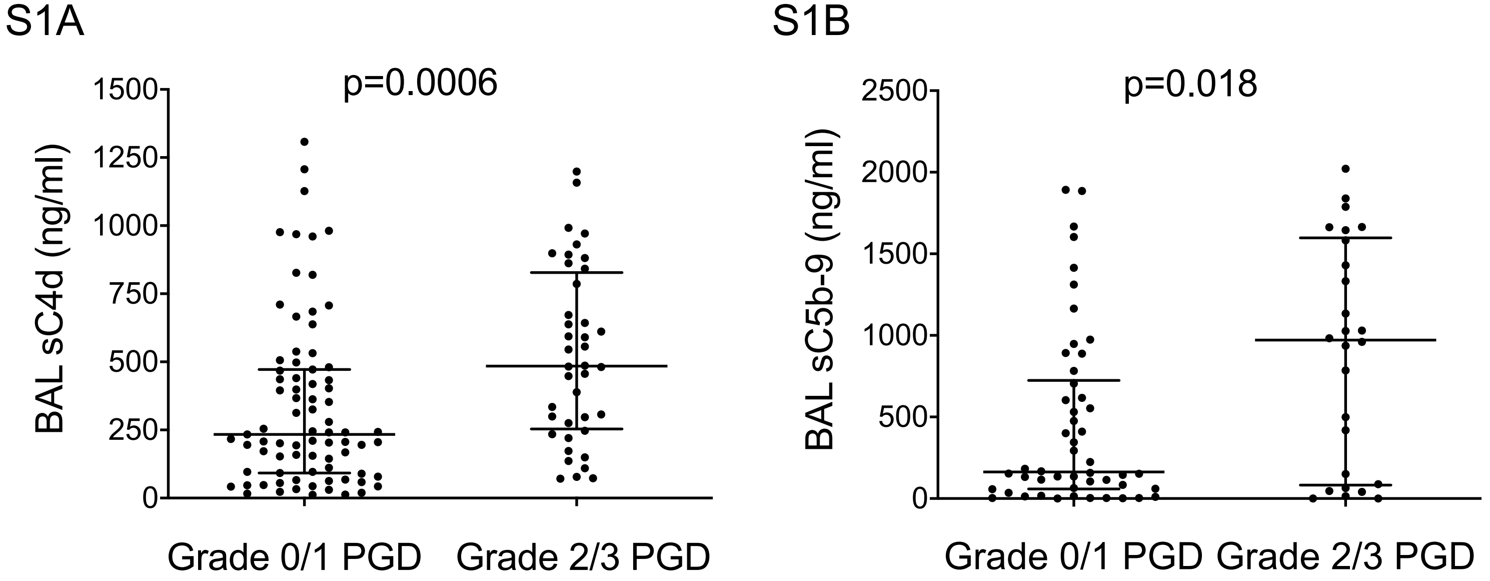

Figure S1. Complement activation is associated with increased PGD severity within the first 24 h of lung transplantation (Penn cohort). Levels of sC4d (A) remained elevated in PGD when the definition of PGD was restricted to those who developed Grade 2 (moderate) or Grade 3 (severe) PGD, as did the levels of sC5b-9 (B). p value is for Mann-Whitney U test.

Supplemental Figure 2

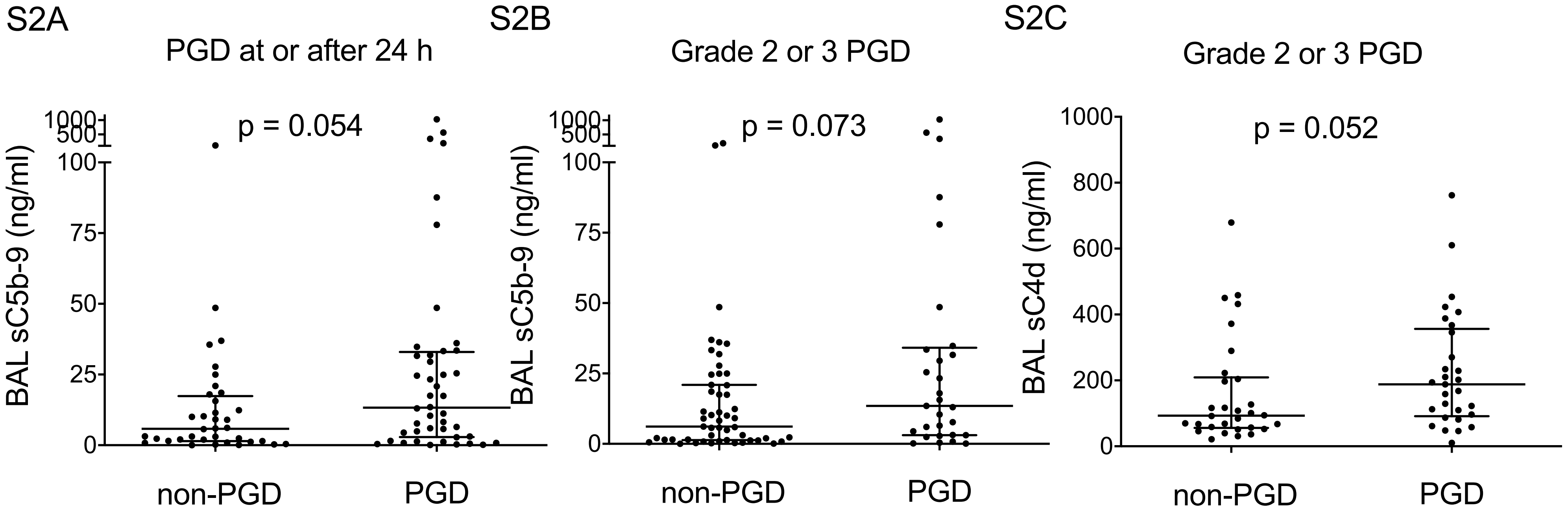

Figure S2. Complement activation is associated with increased PGD severity within the first 24 h of lung transplantation (WUSM cohort). Levels of sC5b-9 remained elevated in subjects with PGD despite restricting the time to event as occurring at or after 24 h (A), or the cohort being defined as subjects with “PGD” if they had only Grade 2 (moderate) or Grade 3 (severe) PGD (B); otherwise they were considered as “non-PGD” subjects. sC4d levels also showed a trend towards significance in those with this restrictive definition compared to ‘non-PGD’ subjects (C). p value is for Mann Whitney U test.

Supplemental Figure 3

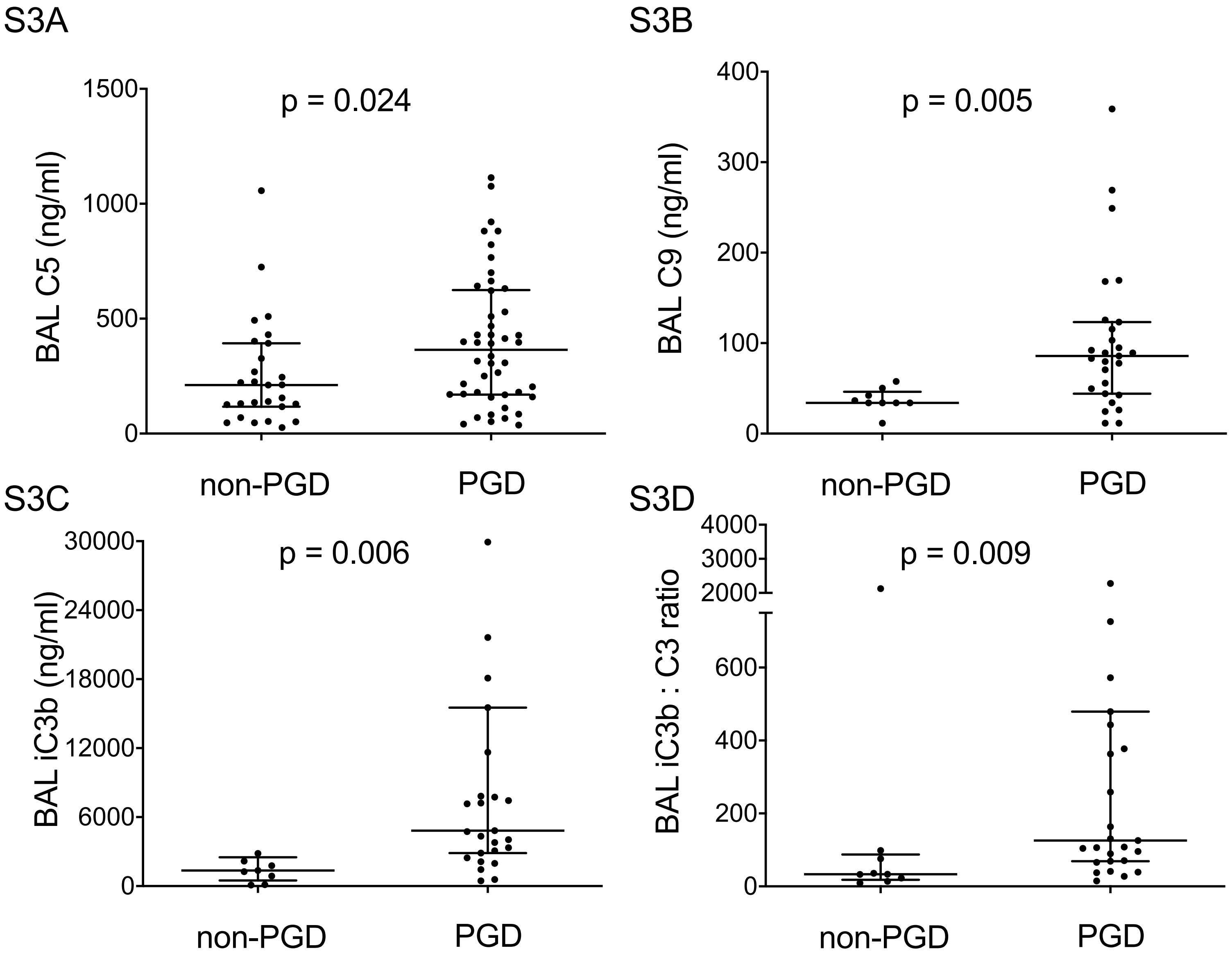

Figure S3C. Subjects with PGD have increased components of the terminal complement cascade locally, as well as markers of complement activation. Levels of C5 (A) and C9 (B), which form components of the terminal complement cascade were elevated in the BAL of subjects with PGD, compared to non-PGD subjects. The generation of iC3b (from C3, C), which indicates cleavage of C3, and thus, complement cascade activation, as well as the iC3b: C3 ratio (D) were also were elevated in subjects with PGD compared to those who did not have PGD. p value is for Mann Whitney U test.

Supplemental Figure 4

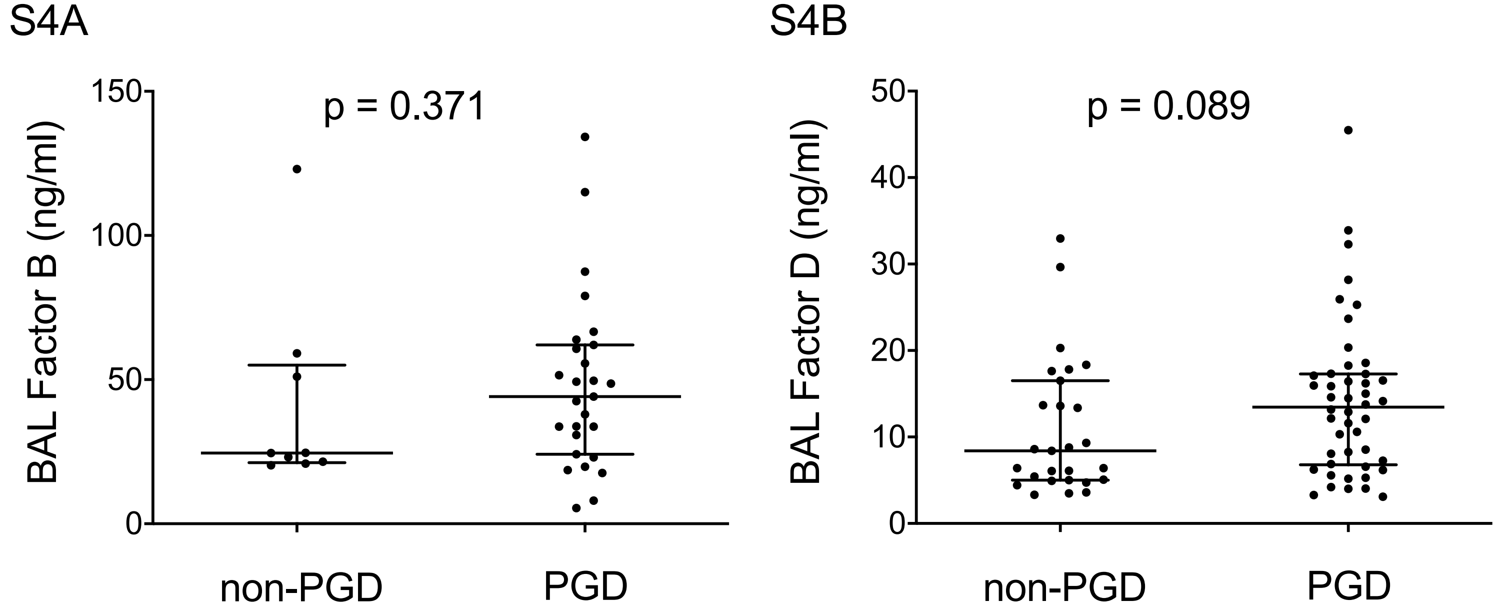

Figure S4. Differences in local meeasurements of alternative pathway components in PGD. There were no differences in levels of total Factor B (A) in the bronchoalveolar lavage (BAL) of subjects with PGD, compared to those without PGD. However, there was a trend in the Factor D (B) levels between the two groups, although it did not reach statistical significance. p values represent Mann Whitney U test.

Supplemental Figure 5

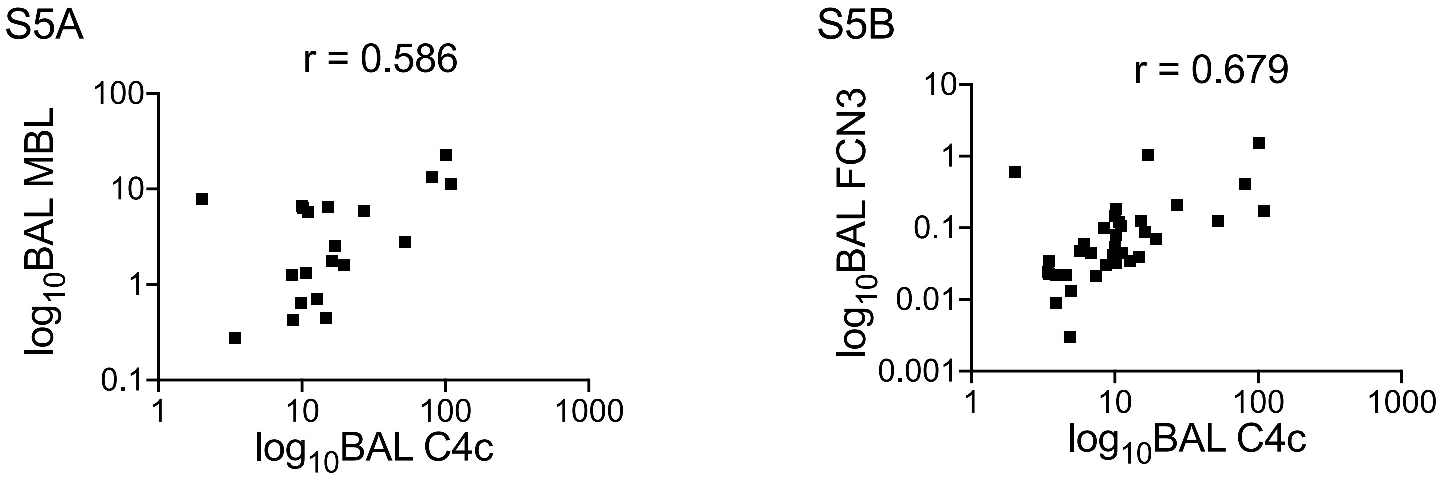

Figure S5. Local markers of lectin pathway activation distinguish subjects with PGD. Levels of mannose-binding lectin (MBL) in the bronchoalveolar lavage (BAL) highly correlated with another marker of complement activation in the BAL (C4c) in the WUSM cohort (A). The levels of ficolin-3 (FCN3, B) also highly correlated with BAL C4c. r represents Spearman's rho coefficient. The axes was expressed in a logarithmic scale for purposes of graphical representation.

Supplemental Figure 6

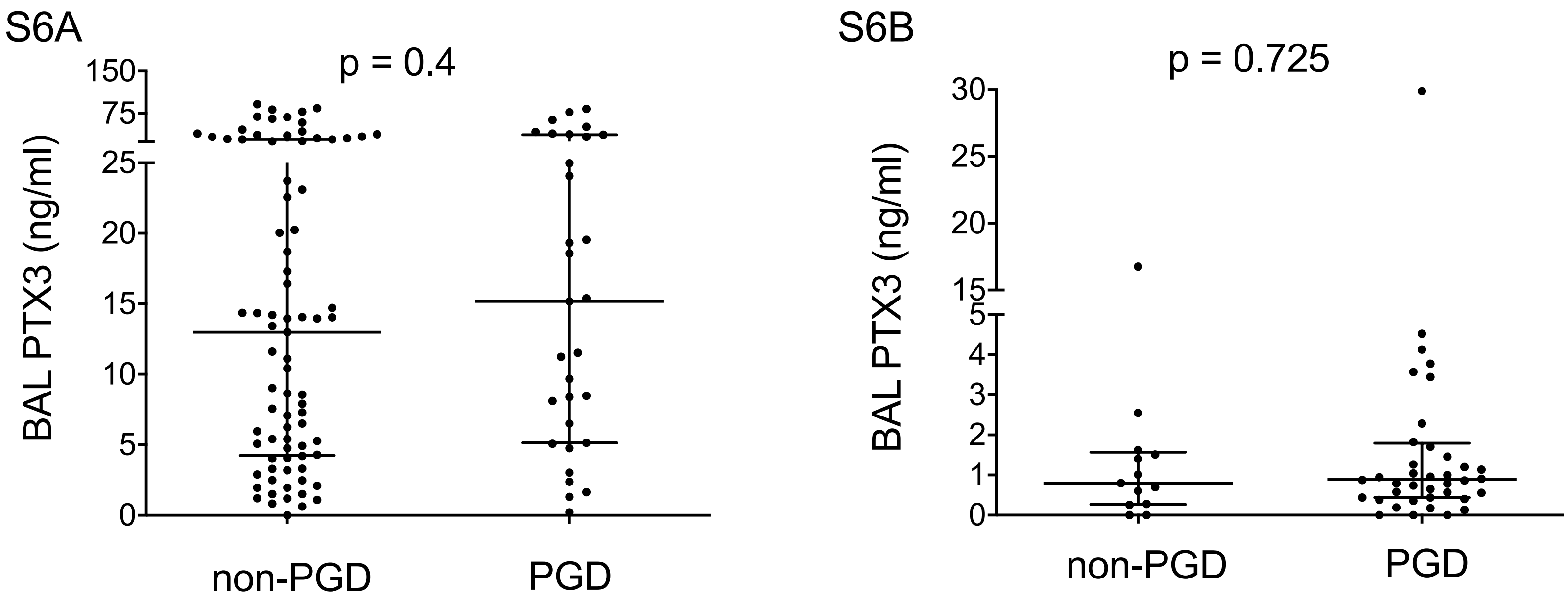

Figure S6. Local levels of long pentraxin 3 (PTX3) were no different in PGD. Levels of PTX3 in the bronchoalveolar lavage (BAL) were no different in subjects wtih PGD versus those who did not develop PGD in either the Penn (A) or WUSM (B) cohort. p value is representative of Mann Whitney U test.
